# Design of Miniaturized 90-Degree Hybrid Coupler with Wide Rejection Band Using Neural Network

**DOI:** 10.3390/mi15050657

**Published:** 2024-05-17

**Authors:** Golshan Mohamadpour, Salman Karimi, Saeed Roshani

**Affiliations:** 1 Department of Electrical Engineering, Lorestan University, Khorramabad 68151, Iran; g_mohamadpour@yahoo.com; 2 Department of Electrical Engineering, Kermanshah Branch, Islamic Azad University, Kermanshah 67189, Iran; s_roshany@yahoo.com

**Keywords:** coupler, size reduction, open-ended stubs, harmonic suppression

## Abstract

In this paper, a 3 dB 90-degree hybrid coupler with size reduction and harmonics rejection was designed. In the proposed coupler structure, four simple low-pass filters (LPFs) were applied. An artificial neural network (ANN) was used to determine the dimensions of the applied LPFs based on EM simulation data. The applied ANN model could also provide the desired LPF parameters, including the cut-off frequency (fc), bandwidth (BW), and insertion loss (IL). Designing an applied LPF involves complex mathematical calculations and simulations to optimize parameters. However, by utilizing neural networks, the design process can be significantly streamlined and automated. Neural networks have the ability to learn complex patterns and relationships within data, making them well suited for optimizing the performance of applied components. The proposed 90-degree hybrid coupler works correctly at 1800 MHz and has a small size of 16.6 mm × 15.15 mm, which provides a 73% size reduction compared to a normal 1800 MHz coupler. The designed coupler not only decreases the circuit size but also provides a wide rejection band from 4.8 GHz to 11.2 GHz, which suppresses the second to sixth harmonics. The insertion loss parameter of this 90-degree hybrid coupler is less than 0.1 dB at the working frequency, which shows the superior performance of the proposed coupler.

## 1. Introduction

As the use of microwaves has expanded in modern communication systems, the primary requirements have become reducing dimensions, eliminating harmonics, and lowering costs while simplifying the design process. Couplers are widely used in microwave devices, such as radar systems [1], UWB circuits [2], OFDM transmission [3], and medical applications [4]. Couplers can be applied to combine or divide power in RF frequencies, with different types. Also, couplers can be incorporated in amplifier and inverter structures [5,6]. The common types of couplers are branch-line, rat-race, and hybrid structures [7,8].

A typical 90-degree hybrid coupler has four branches and combines or divides power with ninety degrees of phase difference [7]. Conventional couplers have very large dimensions. They also pass unwanted harmonics along with the main signal and cause nonlinear effects in the circuit, which is undesirable. These two cases can be called the main disadvantages of conventional couplers [9]. So far, many methods have been used to improve these two defects in couplers, and in many of them, only a few parts of the mentioned problems have been solved, which will be discussed in the following section.

In [10,11], two compact couplers were designed using resonators, with harmonic elimination. The coupler designed in [10] has acceptable performance at the working frequency of 2400 MHz. The above coupler is about 24% smaller than a conventional coupler. Also, the second and third harmonics are removed by the above coupler, which is not impressive. In [11], with resonators applied in a conventional branch-line coupler structure, about a 64% reduction in dimensions was achieved, which was desirable, but the above coupler only removes the third and fifth harmonics, which is not impressive. In [12], a small coupler was designed using coupled transmission lines. This coupler has the same function as a conventional branch-line coupler, but its dimensions were reduced. Unfortunately, in this structure, unwanted harmonics are present in the frequency response and the size reduction is not significant. Also, the application of coupled transmission lines increases the insertion loss. In [13], four resonators were used in a coupler using bent transmission lines. With this method, the dimensions of the coupler were reduced by eighteen percent, and this coupler also eliminates harmonics in a relatively wide frequency band. The obvious feature of the above design is the proper elimination of harmonics, but unfortunately this circuit has a very complex structure. In [14], a coupler with a working frequency of 2.5 GHz and an acceptable size reduction was designed using a T-shaped structure. Unfortunately, the above structure does not provide harmonic elimination. In [15], bent lines and resonators were used to provide a compact coupler with suppressed harmonics. The above coupler has good performance at a frequency of 1.5 GHz, but it has a complex structure. In [16], a compact coupler with harmonic rejection was designed using a defected ground structure (DGS). The DGS technique requires several additional steps that increase fabricating process complexities. In [17], compact couplers with harmonic elimination were designed using lumped elements (inductors and capacitors). DGS and lumped-element methods are not desirable at high frequencies [18].

Neural networks and machine learning algorithms have been widely used to solve different problems and find the nonlinear behaviors behind various structures [19,20,21]. The application of neural networks in the design of a hybrid coupler represents a novel approach to achieving optimal performance in microwave components. Traditionally, the design of RF couplers has involved complex mathematical calculations and simulations to optimize parameters such as the coupling coefficient, isolation, and return loss [22,23,24,25,26,27,28,29,30,31]. However, by utilizing neural networks, the design process can be significantly streamlined and automated. Neural networks have the ability to learn complex patterns and relationships within data, making them well suited for optimizing the performance of microwave components [32]. In some works [33,34,35,36], neural networks have been used to design microstrip filters, couplers, and antennas with optimized performances. In [33], open stubs and T-shaped stubs were used to design a small coupler at 1.8 GHz, which provided second-harmonic rejection. In [33], a neural network was used to find the optimal location of transmission zero. This coupler had a small size, with a 65% size reduction compared with a typical 1.8 GHz coupler.

In [34], a coupler for a wireless system was developed using an artificial neural network (ANN). The applied ANN model was developed based on the back-propagation algorithm to predict parameters and solve for the resonant frequency.

In this work, a compact 90-degree hybrid coupler with a planar structure was proposed at a frequency of 1800 MHz. An ANN model was used to obtain the optimal dimensions of the applied resonator, which resulted in a 73% size reduction compared to a typical 90-degree hybrid coupler and eliminated the second to sixth harmonics. The substrate used to design the proposed coupler was RT/Duroid-5880 with a thickness of 0.508 mm and an ε_r_ of 2.2. The highlights and innovations of the proposed work can be listed as follows:

- The proposed neural network model determines the optimal dimensions of low-pass filters, enhancing the design efficiency of 90-degree hybrid couplers.
- A 73% reduction in the size of the standard coupler was achieved, which could conserve significant space in RF applications.
- The proposed design effectively suppresses the second to sixth harmonics, ensuring cleaner signal transmission and reducing signal interference.

This paper is organized as follows: In Section 2, a short review of existing technologies is provided. This section discusses previous approaches to RF coupler design, highlighting the limitations of the typical coupler. Section 3 provides the methodology and details of the design and implementation of the neural network, including the data preparation, model architecture, and training process. Section 4 provides the results and discussion. It presents the results of the EM simulations, measurements, and neural network model, comparing the predicted and actual outcomes, and discusses the efficacy of the model in the design process. Conclusions are provided in Section 5, which summarizes the key findings and innovations of this research.

## 2. Conventional Branch-Line Coupler Design

**Figure 1:**
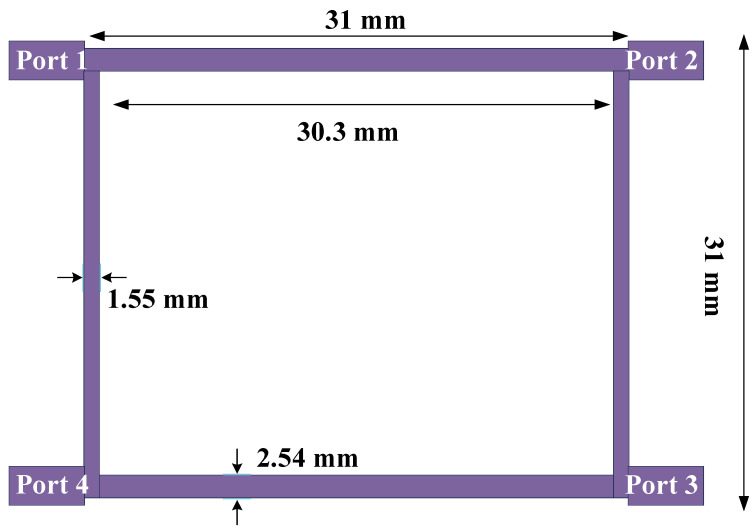
Conventional 90-degree hybrid coupler structure at frequency of 1800 MHz.

**Figure 2:**
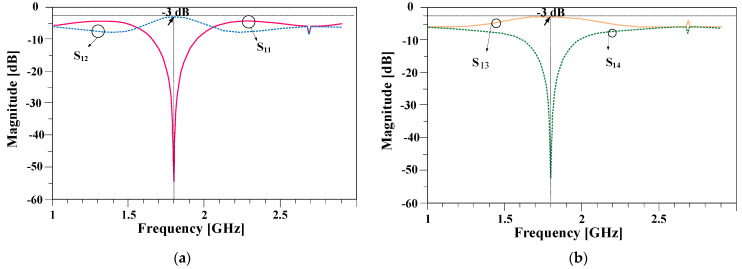
The scattering parameters (**a**) S_11_ and S_12_ and (**b**) S_13_ and S_14_ at working frequency of 1800 MHz.

The structure of a typical 90-degree hybrid coupler at a frequency of 1800 MHz is depicted in Figure 1. As seen in Figure 1, a typical 90-degree hybrid coupler has four long microstrip lines arranged at 90-degree angles (λ/4). The two horizontal lines have an impedance of 35.4 ohms, and the two vertical lines have an impedance of 50 ohms. With the above structure, the scattering parameters of a typical 90-degree hybrid coupler at 1800 MHz are demonstrated in Figure 2.

As seen in Figure 2, for a signal radiated from port 1, at the working frequency, no signal should be re-radiated to port 1 and no part of the signal should be transferred to port 4, so the values of S_11_ and S_14_ should be very small. On the other hand, a signal radiated from port 1 will be divided into two equal parts. Half of the signal will be transferred to the second port, and the other half should be transferred to the third port, so the values of S_13_ and S_12_ should be equal to −3 dB. As shown in Figure 2, the values of S_13_ and S_12_ are −3 dB at the operating frequency of 1800 MHz.

## 3. Proposed Transmission Line with Simple LPF Design

**Figure 3:**
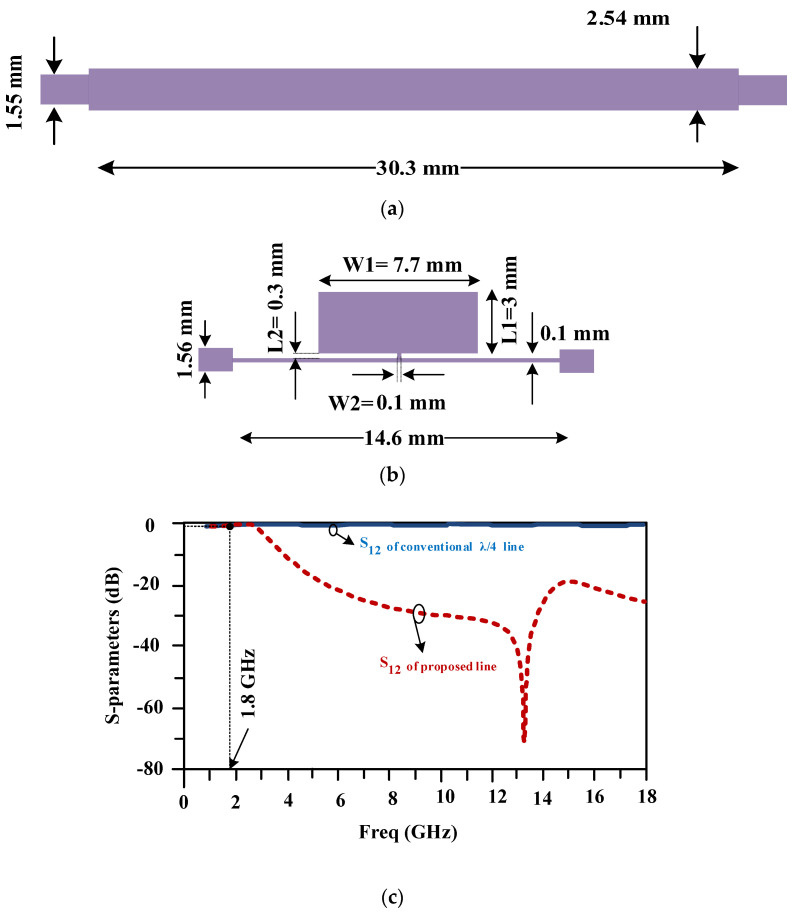
(**a**) The structure of the typical horizontal branch that is used in the normal 90-degree hybrid coupler at the working frequency of 1800 MHz, (**b**) the structure of the designed branch that is used in the designed coupler at a similar working frequency, and (**c**) the frequency responses of the typical and proposed lines.

In a common 90-degree hybrid coupler, four 90-degree lines are used. The structure of the long conventional horizontal line is illustrated in Figure 3a at the working frequency of 1800 MHz. The length of this line with the mentioned substrate is equal to 30.3 mm, and the width of this line is equal to 2.54 mm. Figure 3b shows the structure of the proposed line with a much shorter length of 14.6 mm. Both lines have similar responses at the working frequency of 1800 MHz, which are shown in Figure 3c.

### 3.1. The LC Equivalent Circuit of the Proposed Line

**Figure 4:**
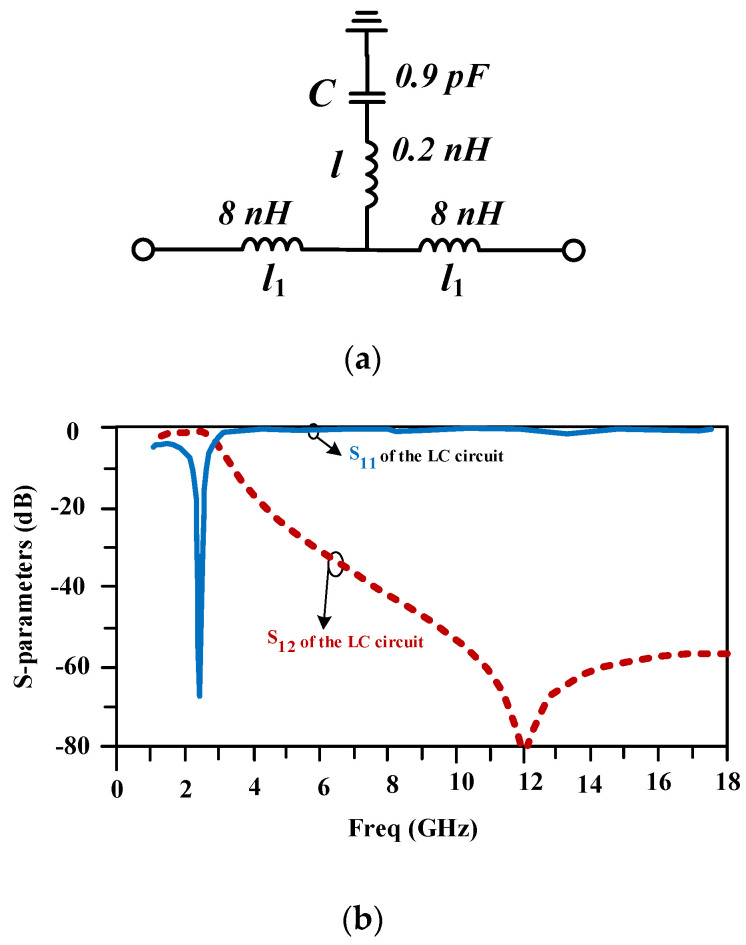
(**a**) The LC equivalent circuit and (**b**) its frequency response for the compact proposed line.

The lumped-element equivalent circuit of the compact proposed line is depicted in Figure 4a. This circuit includes two 8 nH inductors, which are located at the input and output ports, as well as a series inductor and a capacitor with values of 0.2 nH and 0.9 pF. The scattering parameters of the provided lumped-element equivalent circuit are drawn in Figure 4b and are similar to the frequency response of the proposed line.

The extracted transfer function (H(S)) of the LC resonator is provided in (1) as follows:

(1) $$H\left(s\right)\;=\;\frac{1.6\times 10^{-08}s^{4}+4.1\times 10^{09}s^{3}+2.6\times 10^{26}s^{2}+2.4\times 10^{31}s+1.4\times 10^{48}}{s^{4}+1.7\times 10^{18}s^{3}+1.\times 10^{28}s^{2}+4.6\times 10^{38}s+1.4\times 10^{48}}$$

**Figure 5:**
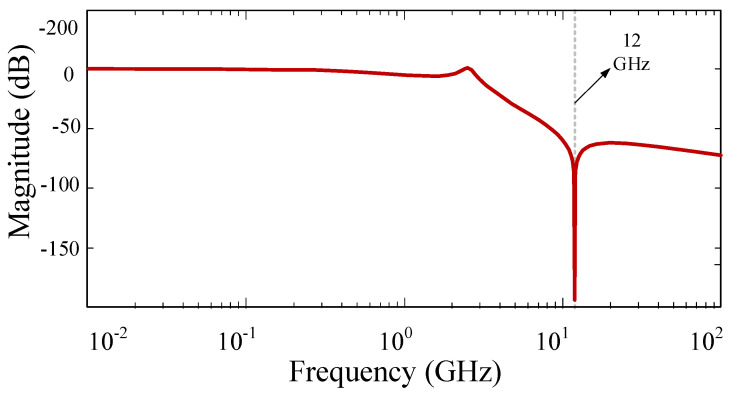
The extracted Bode plot of the provided H(s).

In order to validate the obtained H(s), a Bode plot of this transfer function that was created using Matlab R2022a version software is provided in Figure 5.

The results show that there is good agreement between the extracted Bode plot of the provided H(s), which was created using Matlab R2022a version software, and the S-parameter results of the LC model, which were generated using ADS software (Advanced Design System 2023). This validates the theoretical analysis.

### 3.2. The Structure of the Designed Artificial Neural Network

The designed model utilized a multilayer neural network to predict the parameters of the desired LPF based on the device dimensions. In other words, this model served as a surrogate for predicting LPF parameters. To determine the optimal configuration of the ANN model, various models with various layers were evaluated. The most effective structure consisted of two hidden layers with five and six neurons, offering low complexity.

**Figure 6:**
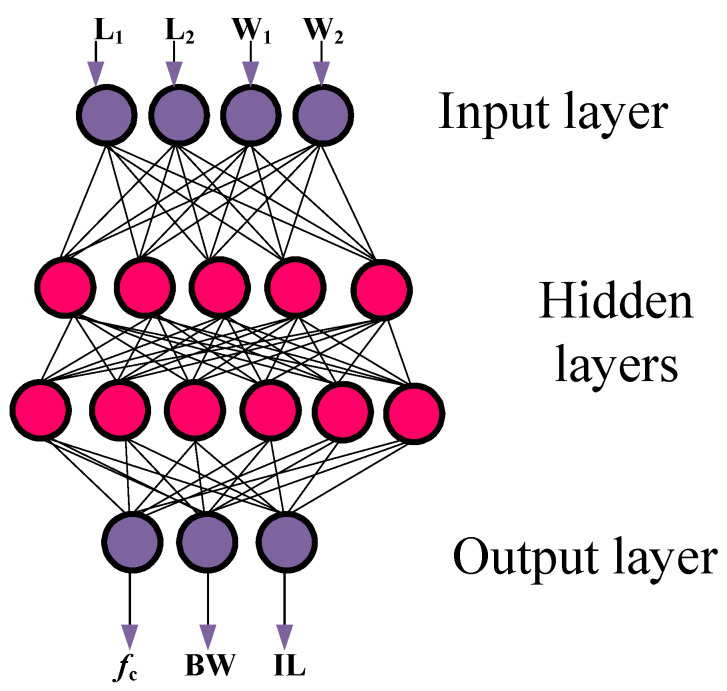
The building blocks of the designed ANN, including two hidden layers with five and six neurons.

Figure 6 illustrates the designed MLP configuration for the applied artificial neural network. In this model, the cut-off frequency (fc), stopband bandwidth (BW), and insertion loss (IL) are considered the outputs of the network. Also, L_1_, L_2_, W_1_, and W_2_, which are shown in Figure 3b, are considered the inputs of the network.

In the design of the proposed miniaturized 90-degree hybrid coupler, a feedforward multilayer perceptron (MLP) network was utilized, structured, and implemented using MATLAB’s newff (new feedforward network) function. The ANN architecture, which was specifically chosen for its efficacy in handling the nonlinear problems typical in electromagnetic simulations, consisted of two hidden layers. The first hidden layer contained five neurons, while the second layer comprised six neurons. They were selected based on empirical trials that were carried out to optimize performance without overfitting. The activation functions used within the network were sigmoid functions, which helped capture the complex mappings between the input dimensions and the filter parameters.

To train the network, backpropagation with a Levenberg–Marquardt optimization, known for its fast convergence in medium-sized networks, was employed. The dataset fed into the ANN comprised parameters extracted directly from electromagnetic simulations conducted to evaluate various LPF configurations. This dataset was split into sets containing 78% for training, 20% for testing, and 2% for validation, ensuring that the model was tested against unseen data to effectively evaluate its generalization capability.

As seen in Figure 6, the parameters of L_1_, L_2_, W_1_, and W_2_, which have main effects on the performance of the proposed filter, were selected as inputs, and the cut-off frequency(fc), stopband bandwidth (BW), and insertion loss (IL) parameters of the designed LPF were considered the output parameters of the proposed ANN model.

For the designed ANN model, 50 samples were used, with 39 samples used to train the ANN model. Then, ten samples were used to test the ANN model. Finally, one sample was applied to validate the designed ANN model.

The related errors of *MRE* and *RMSE* were used to verify the accuracy of the proposed model and were obtained as follows:

(2) $$MRE=\frac{1}{N}\sum_{i=1}^{N}\left|\frac{Y_{Ri}-Y_{Pi}}{Y_{Ri}}\right|$$

(3) $$RMSE=\sqrt{\frac{\sum_{i=1}^{N}{\left(Y_{Ri}-Y_{Pi}\right)}^{2}}{N}}$$

where the *N* in (2) and (3) indicates the number of the dataset, which was 50 for this model. *Y_Pi_* and *Y_Ri_* are the predicted and real outputs of the proposed neural network, respectively.

**Figure 7:**
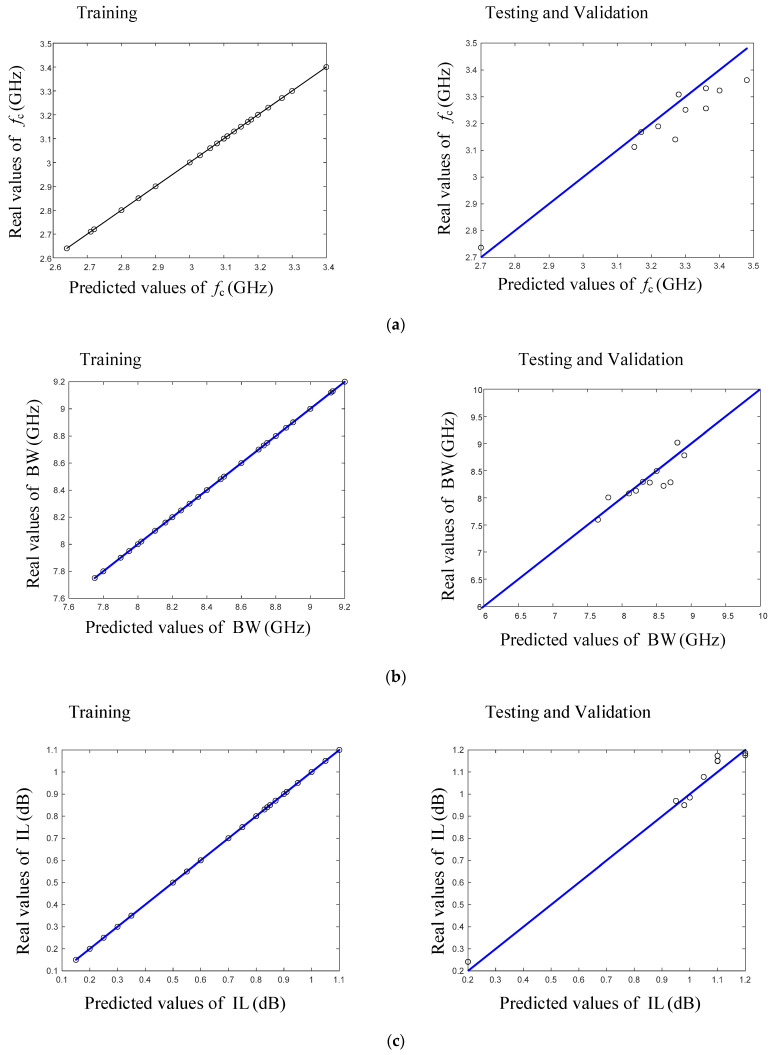
Comparison between predicted and real values of (**a**) *f*_c_ (GHZ), (**b**) BW (GHZ), and (**c**) insertion loss (dB) for testing and training data using proposed ANN model.

The proposed neural network model with two hidden layers, consisting of five and six neurons, was selected as the most accurate model. Figure 7 displays a comparison between the real and predicted values of the output parameters of the applied LPF (fc (GHz), BW (MHz), and insertion loss (dB)) for both the training and testing datasets.

**Figure 8:**
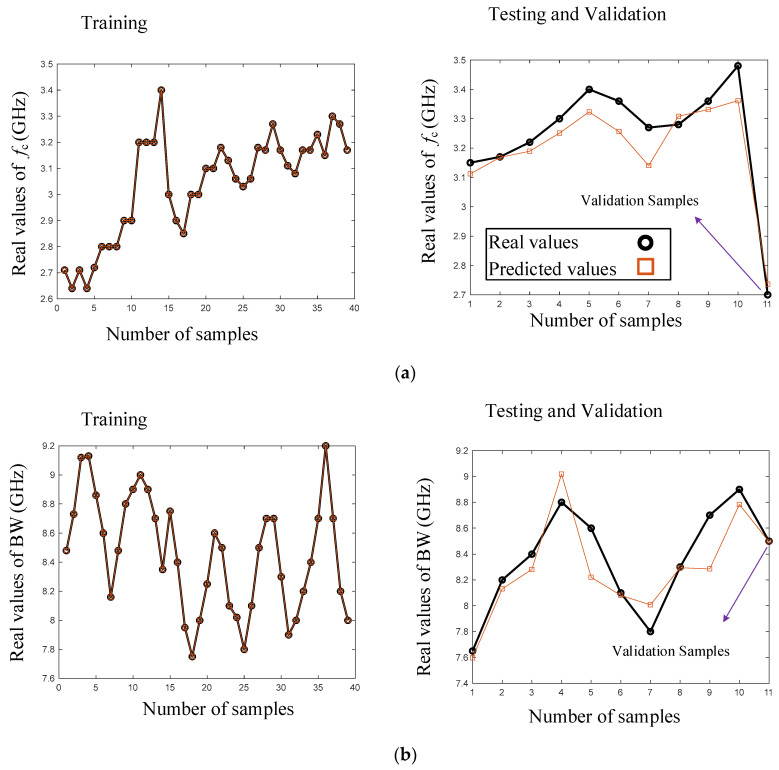

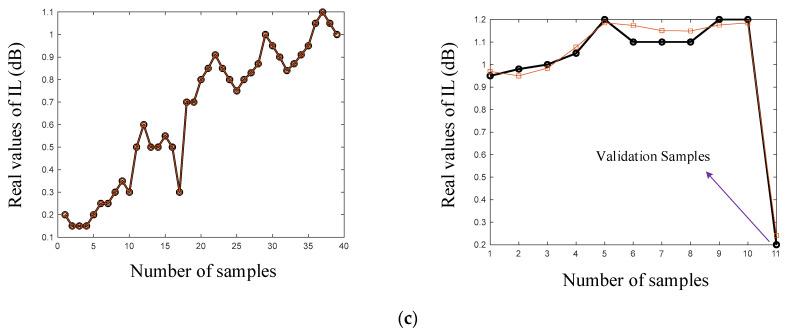
Predicted and real data for training and testing values of (**a**) *f*_c_ (GHZ), (**b**) BW (GHZ), and (**c**) insertion loss (dB) parameters versus numbers of samples in the designed model.

Figure 8 displays a comparison between the predicted and real values of the parameters fc (GHz), BW (MHz), and insertion loss (dB) for both the testing and training datasets in the selected neural network model. The accuracy of the predicted circuit parameters was evident, and the validation sample further confirmed the accuracy of the designed model. As shown in Figure 8, the 39 samples used for the training procedure trained the model accurately. Also, the predicted values of the 10 test samples had acceptable accuracy for all of the LPF outputs. As seen in the figure, the predicted values for insertion loss (IL) and bandwidth (BW) were more accurate than the predicted values for the cut-off frequency (fc). However, the validation sample was used for the design and fabrication of the LPF. The predicted values for the validation sample are also shown in Figure 8 for all of the LPF outputs, which had high accuracy.

The applied dataset and predicted data for the training, testing, and validation procedures are provided in Table 1, including 39 datasets for training, 10 datasets for testing, and 1 dataset for validation. All of the applied samples that were used in the testing, training, and validation processes are listed in Table 1.

The trained model’s predictions of the LPF parameters (cut-off frequency (fc), bandwidth (BW), and insertion loss (IL)) showed high accuracy, with the mean relative errors and root-mean-square errors maintained within the targeted precision limits. These parameters, after being predicted by the ANN, were verified against additional EM simulation results, thereby validating the efficacy and reliability of the neural network approach in streamlining the design process of a 90-degree hybrid coupler.

The outcomes of the designed artificial neural network model are presented in Table 2, demonstrating noteworthy prediction accuracy. As per the information in the table, the model was perfectly trained using the training data.

Table 2 details the performance of the ANN by reporting three critical error metrics: the mean relative error (MRE), root-mean-square error (RMSE), and validation errors for each predicted parameter (cut-off frequency (fc), stopband bandwidth (BW), and insertion loss (IL)). The mean relative error (MRE) and root-mean-square error (RMSE) were crucial in evaluating the accuracy and consistency of the neural network’s predictions. The MRE provided insights into the average magnitude of the errors relative to the actual values, offering a sense of model bias, whereas the RMSE gave a measure of error variability, indicating the prediction stability across the dataset. Low values of MRE and RMSE across the training, testing, and validation datasets, as shown in Table 2, affirmed that the model's predictions were both accurate and consistent. These reported error values substantiate that the ANN model is not only well tuned and robust but also reliable for practical application in designing miniaturized 90-degree hybrid couplers. These findings demonstrate that the neural network effectively captures and predicts the intricate relationships within the RF design parameters, significantly contributing to the field of RF engineering by enabling more efficient and accurate designs.

**Figure 9:**
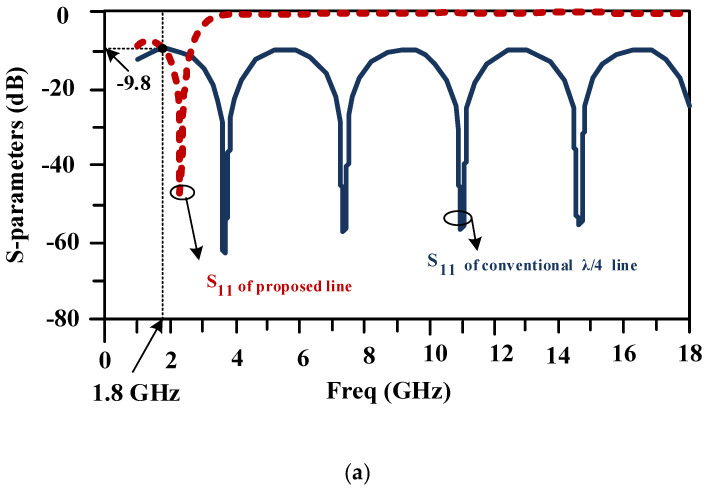

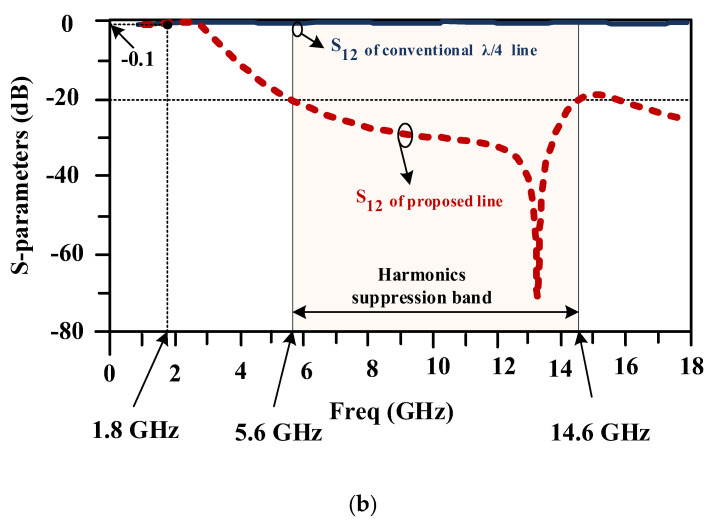
Scattering parameter comparisons between a typical line and the proposed compact line: (**a**) S_11_ and (**b**) S_12_.

A scattering parameter comparison between a typical line and the proposed compact line is illustrated in Figure 9a,b. Both lines have the same response at a frequency of 1.8 GHz. The conventional line has no harmonic elimination, but the proposed line has a wide stopband from 5.6 to 14.6 GHz, which shows the proper performance of the above line with much smaller dimensions. As seen in the figure, the proposed line acts as an LPF with a cut-off frequency of 2.7 GHz, which allows signals from DC to 2.7 GHz to pass and suppresses signals at higher frequencies. As seen in Figure 9, the value of S_11_ in the pass band is less than 9 dB and the value of S_21_ is near 0 dB (0.1 dB) for both the conventional and proposed lines, which is acceptable.

**Figure 10:**
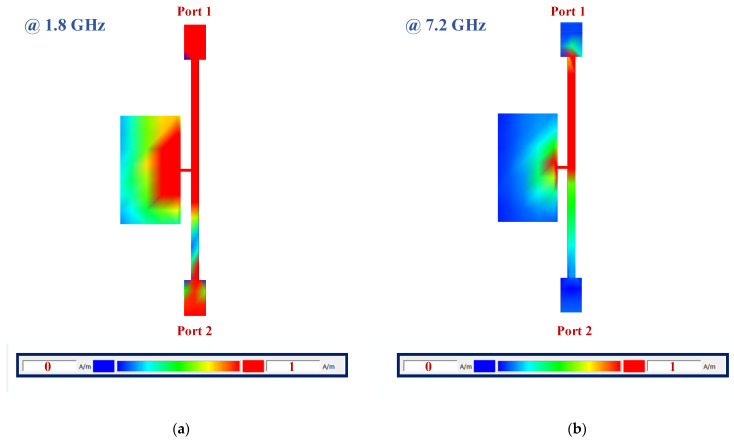
The current distributions of the applied low-pass filter at (**a**) 1.8 GHz (fundamental frequency of the coupler), which is located in the pass band, and (**b**) 7.2 GHz (frequency of the 4th harmonic of the coupler), which is located in the stopband.

Figure 10 depicts the current distributions of the applied low-pass filter at 1.8 GHz and 7.2 GHz. The applied LPF has a cut-off frequency of 2.7 GHz, while the designed coupler is working at 1.8 GHz. As shown in Figure 10a, the signal coming from port 1 correctly passes to port 2. Also, the proposed LPF provides a wide rejection band from 5.6 to 14.6 GHz with more than 20 dB of attenuation. As seen in Figure 10b, the signal coming from port 1 at 7.2 GHz, which is located in the stopband, does not pass to port 2.

## 4. Proposed 90-Degree Hybrid Coupler

**Figure 11:**
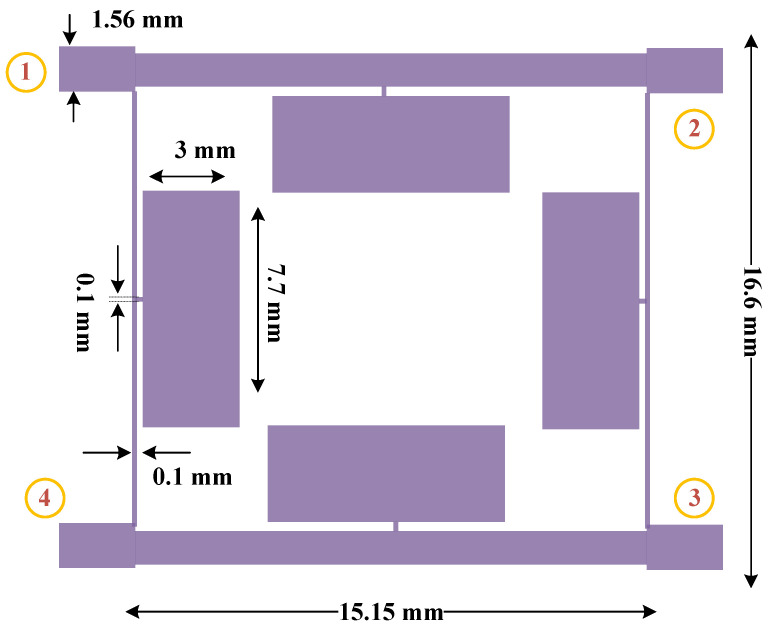
The configuration of the proposed 90-degree hybrid coupler at the working frequency of 1800 MHz.

The structure of the proposed 90-degree hybrid coupler is illustrated in Figure 11. The final dimensions of the designed 90-degree hybrid coupler are 16.6 mm × 15.15 mm, which provides a size reduction of 73% compared to the dimensions of a common coupler (31 mm × 31 mm) at the same frequency of 1800 MHz.

**Figure 12:**
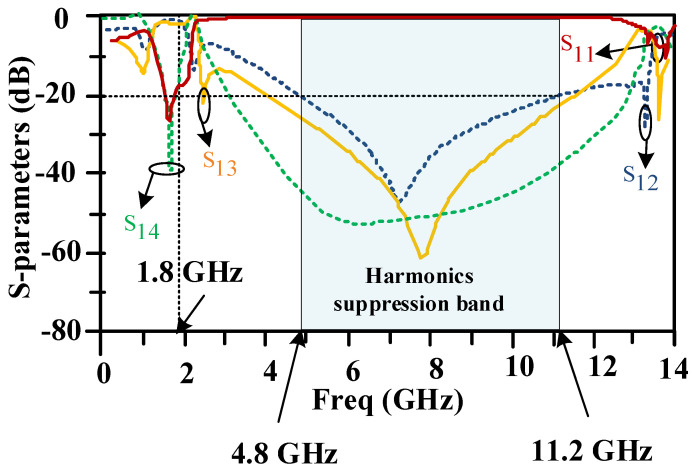
Scattering parameters of the designed 90-degree hybrid coupler at a working frequency of 1800 MHz.

Figure 12 shows the simulated frequency response of the proposed 90-degree hybrid coupler. The designed 90-degree hybrid coupler has a good response at the working frequency of 1800 MHz, and the insertion loss is 0.1 dB. Also, the designed coupler provides a wide cut-off bandwidth in a range from 4.8 GHz to 11.2 GHz with an attenuation level exceeding 20 decibels.

**Figure 13:**
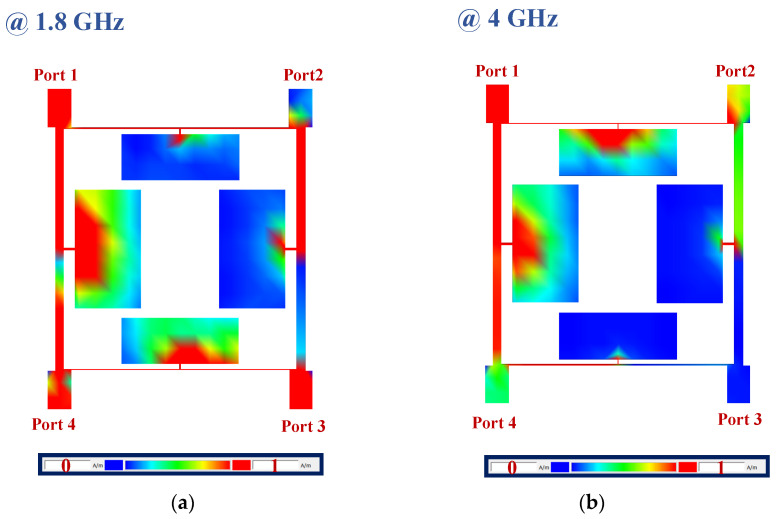
The current distributions of the proposed coupler at (**a**) 1.8 GHz, which is the working frequency of the coupler, and (**b**) 4 GHz, which is located in the rejected band.

In Figure 13, the current distributions of the designed 90-degree hybrid coupler at 1800 MHz and 4 GHz are depicted. The proposed 90-degree hybrid coupler operates at 1800 MHz and provides a rejection band from 4.8 to 11.2 GHz. As shown in Figure 13a, the signal coming from port 1 at 1800 MHz correctly passes to port 2 and port 3, while no signal is transmitted to port 4. Additionally, as seen in Figure 13b, the signal coming from port 1 at 4 GHz, which is located in the stopband, does not pass to port 2 or port 3.

**Figure 14:**
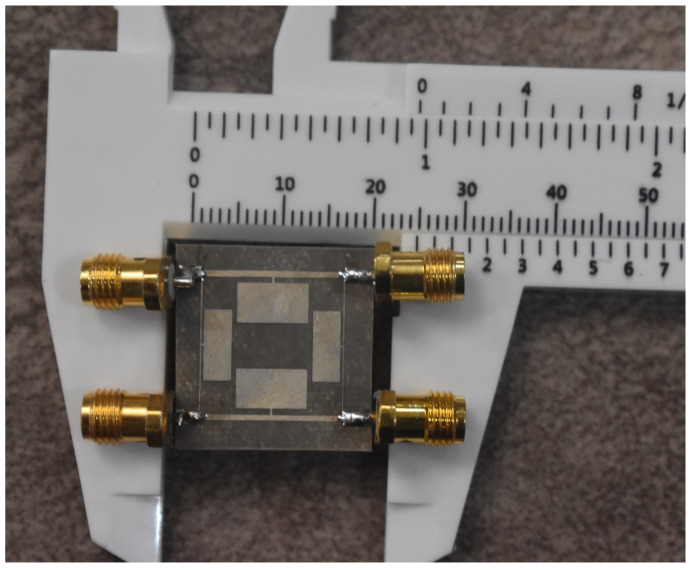
Photo of the designed 90-degree hybrid coupler at a frequency of 1800 MHz.

The designed 90-degree hybrid coupler was fabricated on an RT duroid 5880 Rogers substrate, and a photo of the designed coupler is presented in Figure 14. The final dimensions of the coupler are only 16.6 mm × 15.15 mm, which is a size reduction of 73% compared to the dimensions of a common coupler (31 mm × 31 mm) at a working frequency of 1800 MHz. The simple planar structure of the microstrip coupler allows for easy fabrication without the need for additional processes.

**Figure 15:**
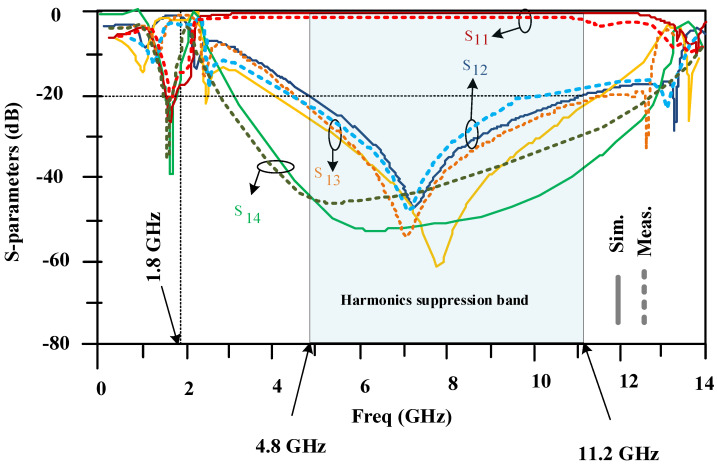
Measured and simulated frequency responses of the proposed coupler.

The measured and simulated frequency responses of the proposed 90-degree hybrid coupler are presented in Figure 15. There is good agreement between the simulations and measurements. The measurements confirm the simulations. The proposed coupler has good performance at 1.8 GHz. Also, the proposed coupler provides a wide harmonics rejection band in a frequency range from 4.8 GHz to 11.2 GHz.

**Figure 16:**
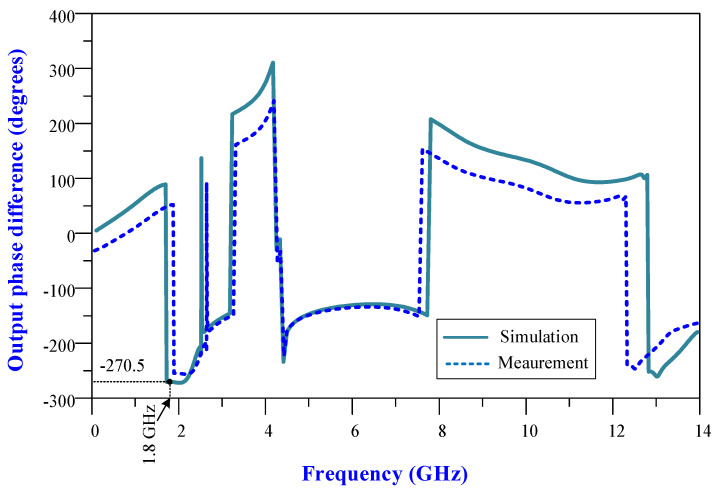
Measured and simulated output phase differences of the designed coupler.

The measured and simulated output phase curves of the 90-degree hybrid coupler are illustrated in Figure 16. The output phase difference at 1.8 GHz is −270.5 degrees, which is 0.5 degrees out of phase.

In Table 3, the performance of the designed 90-degree hybrid coupler is compared with those of couplers from some similar works. In the comparison, the advantages of the 90-degree hybrid coupler in terms of its low losses, small size, and wide rejection band can be clearly seen.

## 5. Conclusions

In this paper, a small 90-degree hybrid coupler was designed using open-ended lines at a working frequency of 1800 MHz. The proposed 90-degree hybrid coupler has a very simple structure and is 73% smaller than a typical 90-degree hybrid coupler. The designed coupler has very good performance at the working frequency, and its insertion loss is less than 0.1 dB. Also, the proposed coupler provides a wide stopband at higher frequencies and removes unwanted signals from 4.8 GHz to 11.2 GHz with more than 20 dB of attenuation, which includes the second to sixth harmonics. The above-mentioned coupler is suitable for wireless communication applications. In the proposed coupler structure, four simple low-pass filters (LPFs) are utilized. An artificial neural network (ANN) was used to determine the dimensions of the applied LPFs based on EM simulation data. Designing an applied LPF involves complex mathematical calculations and simulations to optimize parameters. However, by utilizing neural networks, the design process can be significantly streamlined and automated.

## Figures and Tables

**Table 1:** The applied dataset and predicted data for the training, testing, and validation procedures.

		Input				Output		
		W1 (mm)	L1 (mm)	W2 (mm)	L2 (mm)	Fc (GHz)	Stopband Bandwidth (GHz)	IL @ 1800 MHz (dB)
1	Training Values of Design Parameters	7.9	3	0.1	0.3	2.71	8.48	0.2
2		7.9	3.2	0.1	0.3	2.64	8.73	0.15
3		7.9	3.2	0.2	0.3	2.71	9.12	0.15
4		7.9	3.2	0.2	0.4	2.64	9.13	0.15
5		7.5	3.2	0.2	0.4	2.72	8.86	0.2
6		7.5	3	0.2	0.4	2.8	8.6	0.25
7		7.5	3	0.1	0.4	2.8	8.16	0.25
8		7.5	3	0.1	0.3	2.8	8.48	0.3
9		7.5	3	0.1	0.2	2.9	8.8	0.35
10		7.2	3	0.1	0.2	2.9	8.9	0.3
11		7.2	2.8	0.1	0.2	3.2	9.0	0.5
12		7.0	2.8	0.1	0.2	3.2	8.9	0.6
13		7.0	2.8	0.1	0.3	3.2	8.7	0.5
14		7.0	2.8	0.1	0.4	3.4	8.35	0.5
15		7.0	2.8	0.2	0.4	3.0	8.75	0.55
16		7.0	2.8	0.2	0.5	2.9	8.4	0.5
17		7.0	2.8	0.1	0.5	2.85	7.95	0.3
18		7.0	2.5	0.1	0.5	3.0	7.75	0.7
19		7.0	2.5	0.1	0.4	3.0	8.0	0.7
20		7.0	2.5	0.1	0.3	3.1	8.25	0.8
21		7.0	2.5	0.1	0.2	3.1	8.6	0.85
22		6.8	2.5	0.1	0.2	3.18	8.5	0.91
23		6.8	2.5	0.1	0.3	3.13	8.1	0.85
24		6.8	2.5	0.1	0.4	3.06	8.02	0.8
25		6.8	2.5	0.1	0.5	3.03	7.8	0.75
26		6.8	2.5	0.2	0.5	3.06	8.1	0.8
27		6.8	2.5	0.2	0.4	3.18	8.5	0.83
28		6.8	2.5	0.2	0.3	3.17	8.7	0.87
29		6.8	2.4	0.1	0.2	3.27	8.7	1.0
30		6.8	2.4	0.1	0.3	3.17	8.3	0.95
31		6.8	2.4	0.1	0.4	3.11	7.9	0.90
32		6.8	2.4	0.1	0.5	3.08	8.0	0.84
33		6.8	2.4	0.2	0.5	3.17	8.2	0.87
34		6.8	2.4	0.2	0.4	3.17	8.4	0.91
35		6.8	2.4	0.2	0.3	3.23	8.7	0.95
36		6.8	2.4	0.2	0.2	3.15	9.2	1.05
37		6.5	2.4	0.1	0.2	3.3	8.7	1.1
38		6.5	2.4	0.1	0.3	3.27	8.2	1.05
39		6.5	2.4	0.1	0.4	3.17	8.0	1.0
40	Testing Values of Design Parameters	6.5	2.4	0.1	0.5	3.15	7.65	0.95
41		6.5	2.4	0.2	0.5	3.17	8.2	0.98
42		6.5	2.4	0.2	0.4	3.22	8.4	1.0
43		6.5	2.4	0.2	0.3	3.3	8.8	1.05
44		6.5	2.2	0.1	0.3	3.4	8.6	1.2
45		6.5	2.2	0.1	0.4	3.36	8.1	1.1
46		6.5	2.2	0.1	0.5	3.27	7.8	1.1
47		6.5	2.2	0.2	0.5	3.28	8.3	1.1
48		6.5	2.2	0.2	0.4	3.36	8.7	1.2
49		6.5	2.2	0.2	0.3	3.48	8.9	1.2
50	Validation Values of Design Parameters	7.7	3	0.1	0.3	2.7	8.5	0.2

**Table 2:** Final obtained values of the applied neural network model.

	*f*_c_ (GHz) Errors			BW (GHz) Errors			IL (dB) Errors		
	Training	Testing	Valid.	Training	Testing	Valid.	Training	Testing	Valid.
MRE	1.71 × 10^−11^	0.0181	0.0135	7.45 × 10^−8^	0.0189	6.09 × 10^−4^	6.27 × 10^−9^	0.0295	0.2056
RMSE	7.47 × 10^−11^	0.0734	0.0364	4.18 × 10^−6^	0.2101	0.0052	2.37 × 10^−9^	0.0370	0.0411

**Table 3:** Performance comparison of the proposed 90-degree hybrid coupler and couplers from similar works.

Reference	Frequency (GHz)	Size Reduction (%)	Insertion Loss (dB)	Harmonic Suppression
[17]	0.9	64	0.3	3rd and 5th harmonics
[33]	1.8	65	0.4	2nd harmonic
[37]	0.9	76.6	0.9	4th harmonic
[38]	2.4	53	N/A	No harmonic suppression
[39]	1	71	0.8	4th harmonic
[40]	1.39	78	0.4	3rd harmonic
[41]	1	63	0.3	3rd harmonic
[42]	2.1	63	0.9	3rd harmonic
[43]	2	30	0.5	3rd harmonic
[44]	1	50	0.33	No harmonic suppression
Proposed Coupler	1.8	73	0.1	2nd to 6th harmonics

## Data Availability

All data from this study are mentioned in this article.
